# Supplementary material for: Permeability of membranes in the liquid ordered and liquid disordered phases
Source: Nat Commun. 2019 Dec 9;10:5616. doi: 10.1038/s41467-019-13432-7 (PMC6901538; doi:10.1038/s41467-019-13432-7)
Supplement: Supplementary file 1 — Supplementary Information [file 41467_2019_13432_MOESM1_ESM.pdf]

SUPPLEMENTARY INFORMATION

Permeability of Membranes in the Liquid Ordered and Liquid Disordered Phases

An Ghysels,[1] Andreas Krämer,[2] Richard M. Venable,[2] Walter E. Teague Jr.,[3] Edward Lyman,[4] Klaus Gawrisch,[3] and Richard W. Pastor[2]

[1]Center for Molecular Modeling, Ghent University, Technologiepark 46, 9052 Gent, Belgium

[2]Laboratory of Computational Biology, National Heart Lung Blood Institute, National Institutes of Health, Bethesda, Maryland 20892, USA

[3]Laboratory of Membrane Biochemistry and Biophysics, National Institute on Alcohol Abuse and Alcoholism, National Institutes of Health, Bethesda, Maryland 20892, USA

[4]Department of Physics and Astronomy and Department of Chemistry and Biochemistry, University of Delaware, Newark, 19716 Delaware, USA

Supplementary Notes

In the main document and supplementary information, the term "L_o and L_d phases" refers to the liquid ordered phase and liquid disordered phase of DPPC/DOPC/chol, respectively, for which the simulation details and composition are given in Supplementary Table 1, unless noted otherwise. It will be stated explicitly when the discussed ordered or disordered phases contains PSM. Supplementary Table 3 gives the system details of the Anton simulations.

Supplementary Figures 3 and 4 show simulation snapshots of oxygen and water entering membranes, respectively.

Supplementary Figures 6, 7 and 8 show structural membrane properties. Various structural properties of the simulated L_o and L_d phases at 298 K are compared to simulations of one-component (homogeneous) membranes: (1) DOPC at 298 K ($L_{\alpha,298}$) and (2) DPPC at 323 K ($L_{\alpha,323}$). Pure DPPC is a gel at 298 K, hence the choice for the higher temperature of 323 K, to ensure that homogeneous DPPC has liquid disordered behavior.

Supplementary Methods

1. Oxygen concentrations and force field errors

Oxygen concentrations in water and membrane phases corresponding to the EPR experiments (membrane vesicles in equilibrium with 100% O_2 atmosphere at 1 atm pressure or air at 1 atm pressure) can be estimated for the number of water molecules and dimensions of the simulation systems listed in Supplementary Table 1 from: (1) the experimental value of the solubility of O_2 in water (1.298×10^{-3} mol/l-atm at 295 K)[1], (2) the membrane/water partition coefficient for O_2 (3.2 for egg-yolk phosphatidylcholine at 295 K;[2] see ref. 3 for partition coefficients of other lipids), and (3) the molecular volume of water (30 A^3). Each water molecule is assumed to take up the same volume, and the remaining volume of the simulation cell is assigned to the membrane. Estimated numbers of oxygen atoms in water and membranes exposed to a pure oxygen

atmosphere at a pressure of 1 atm are reported in Supplementary Table 6. Assuming that the solubility of oxygen in water increases linearly with oxygen pressure and that the membrane/water partition coefficient is independent of oxygen pressure, simulated systems with 50 O_2 are under 33 atm and 34 atm for the L_o and L_d phases, respectively. Values for air (21% O_2) are about 7 atm for both L_o and L_d . The membrane thickness h is estimated by subtracting the volume taken up by all of the water molecules from the volume of the simulation cell. The resulting $h/2$ are smaller than those used in the counting method and BA method for the permeability calculations (Table 1 of the main text). This is because the latter are based on dividing surfaces above the membrane surface where the free energy profiles become flat (Fig. 2 of main text) and consequently include some of the waters in the system.

A comparison of solubility of oxygen in hexadecane and water expressed as hexadecane/water partition coefficient $K_{w\to h}$ obtained in experiment and simulation allows evaluation of simulation force fields. $K_{w\to h}$ is defined as the ratio of O_2 solubility in hexadecane[1] (10.64 x 10^{-3} mol/l-atm) and water (1.298 x 10^{-3} mol/l-atm) equal to 8.19 at 295 K (experiment). Supplementary Figure 1 shows the PMF of oxygen in a hexadecane/water slab according to MD simulations with the CHARMM36 force field at three temperatures: (1) 800 ns simulation at 295 K (the experimental target temperature), (2) 800 ns simulation at 298 K (the temperature of bilayer simulations in the main text), and (3) 800 ns simulation at 310 K, with (2) and (3) as reported previously[4]. $\Delta G_{w\to h}^{295} = RT \ln K_{w\to h}$ = 1.75 kcal/mol, yielding $K_{w\to h}$ (sim) = 18.5; i.e., the partition coefficient from the simulation is about two-fold larger than the experimental one. This result implies that the membrane/water partition coefficient of oxygen from simulation overestimates that of experiment by approximately a factor of 2. This overestimate is expected to carry over for membranes as well because oxygen mostly resides in the hydrocarbon region.

2. EPR experiments

Transverse relaxation rate (R_2) analysis:

Linewidth was measured as peak-to-peak distance of the first derivative of the $M_I = 0$ resonance.

L_o (N_2): 2.71 ± 0.025 G

L_o(O_2): 2.97 ± 0.025 G contribution from oxygen: 0.26 ± 0.05 G

L_d (N_2): 2.10 ± 0.025 G

L_d (O_2): 2.51 ± 0.025 G contribution from oxygen: 0.41 ± 0.05 G

$R_{2,Ld}/R_{2,Lo} \propto 0.41/0.26 = 1.6 \pm 0.5$

Longitudinal relaxation rate (R_1) analysis:

Power saturation curves were measured as peak-to-peak height of the $M_I = 0$ (center) resonance as shown in Fig. 6 of the main document. The parameter $P_{1/2}$ was determined by fitting Eq. (8) to the experimental data points with I_0 and $P_{1/2}$ as fit parameters[5,6]. The fit was executed using a Marquardt-Levenberg algorithm (SigmaPlot, Systat Software, Inc.). The lines through data points indicate the quality of the fit in Fig. 7. It can be shown that $P_{1/2} \propto R_1 R_2$. Division by $P_{1/2}$ by the linewidth of the $M_I = 0$ resonance yields a parameter proportional to R_1. The formula assumes that saturation of the $M_I = 0$ resonance follows a homogeneous saturation limit which was confirmed experimentally. The uncertainties listed below are standard errors from the fits.

L_o (N_2):	$P_{1/2}$ = 53.2 ± 1.7 mW	Lw = 2.98 ± 0.025 G	$P_{1/2}$/Lw = 17.8 ± 0.7
L_o(air):	$P_{1/2}$ = 124.3 ± 6.6 mW	Lw = 3.04 ± 0.025 G	$P_{1/2}$/Lw = 40.9 ± 2.5
L_d (N_2):	$P_{1/2}$ = 31.6 ± 0.8 mW	Lw = 2.05 ± 0.025 G	$P_{1/2}$/Lw = 15.4 ± 0.5
L_d (air):	$P_{1/2}$ = 128.1 ± 7.4 mW	Lw = 2.16 ± 0.025 G	$P_{1/2}$/Lw = 59.3 ± 4.1

$P_{1/2}$/Lw contribution from oxygen L_0 = 23.1 ± 3.3

$P_{1/2}$/Lw contribution from oxygen L_d = 43.9 ± 4.6

$R_{1,Ld}/R_{1,Lo} = 1.90 \pm 0.5$

Supplementary Tables

Supplementary Table 1. Component numbers (mole fraction in parentheses), cell areas (*A*) and heights (*H*), and trajectory lengths (T_{run}) used for analysis.

property	L_o	L_d
DPPC	280 (0.55)	116 (0.30)
DOPC	76 (0.15)	240 (0.62)
cholesterol	156 (0.30)	44 (0.08)
waters	9999	12185
oxygen	50	50
ion pairs[a]	31	30
A (nm^2)	108.8	119.5
H (nm)	7.533	7.054
T_{run} (μs)	1.14[b]	0.95

[a] Na^+ and Cl^-
[b] 1.12 μs analyzed for O_2

Supplementary Table 2. Number of crossings and crossing rate *r* for oxygen and water evaluated using the counting method. The 95% confidence intervals in parentheses were obtained assuming a Poisson distribution.

Permeant	# *crossings*		r (10^{-3} nm^{-2} ns^{-1})	
	L_o	L_d	L_o	L_d
O_2	262 (231-294)	466 (424-509)	1.98 (1.74-2.22)	3.44 (3.13-3.75)
water	11 (5-18)	69 (53-86)	0.0815 (0.037-0.133)	0.0508 (0.0391-0.0634)

Supplementary Table 3. Component numbers, temperature T, cell areas A and heights H, and trajectory lengths used for analysis T_{run} for simulations without oxygen carried out on Anton[7,8]. Saturated refers to DPPC and PSM, unsaturated to DOPC and POPC, and ions are sodium and chloride.

	DPPC/DOPC/chol		PSM/DOPC/chol		PSM/POPC/chol	
property	L_o	L_d	L_o	L_d	L_o	L_d
saturated lipids	280	116	362	84	342	128
unsaturated	76	240	14	460	44	386
cholesterol	156	44	184	30	174	44
waters	9999	12185	18684	23261	22848	20487
ion pairs	31	30	0	0	0	0
T (K)	298	298	295	295	295	295
A (nm^2)	104.9	114.3	113.4	179.8	113.4	153.5
H (nm)	7.643	7.219	9.577	7.734	10.722	8.125
T_{run} (μs)	5.05	2.02	0.95	1.14	9.08	0.81

Supplementary Table 4. Component surface areas (Å^2/lipid) at 298 K unless otherwise noted. Source data are provided as a Source Data file.

component	L_o	L_d	L_α
DPPC	48.8	61.8	63.0[a]
DOPC	51.5	64.1	68.9
cholesterol	26.8	30.6	-

[a]at 323 K

Supplementary Table 5. Entrance and escape times for oxygen and water to and from the midplane. Standard errors in parentheses. Source data are provided as a Source Data file.

permeant	phase	τ_{entr} (ns)	τ_{esc} (ns)
Oxygen	L_o	2.0 (0.09)	62.3 (7.5)
	L_d	1.0 (0.02)	38.3 (4.0)
	L_α (DOPC)[a]	1.0 (0.01)	40.0 (5.0)
Water[b]	L_o	0.67 (0.1)	1.23 (0.3)
	L_d	0.41 (0.2)	0.70 (0.3)
	L_α (DOPC)[c]	0.61 (0.2)	0.60 (0.1)

[a]from ref.9; $h/2$ = 25 Å
[b]from the planes defined by phosphate groups; $h/2$ = 24.0 Å for L_o, 20.4 Å for L_d, and 19.2 Å for L_α
[c]from ref. 10

Supplementary Table 6. Calculated number of oxygen molecules in water and membranes for a system with dimensions and particle numbers as listed in Supplementary Table 1 assuming a pure oxygen atmosphere at a pressure of 1 atm, an oxygen solubility in water of 1.298 x 10^{-3} mol/l-atm at 295 K and a membrane/water partition coefficient of 3.2. The membrane thickness h here is calculated by subtracting the volume taken up by all of the water molecules from the volume of the simulation cell.

region	L_o	L_d
water	0.23	0.29
membrane	1.30	1.19
total	1.53	1.48
$h/2$ (Å)	23.9	20.0

Supplementary Table 7. Concentration of permeants in water (c_w) and membrane (c_m) and partition coefficient *K(h)* evaluated with $h/2$ = 29.38 Å and 26.39 Å for the L_o and L_d phases, respectively. Twice the standard error over four replicates in parenthesis. Source data are provided as a Source Data file.

Permeant	c_w (nm^{-3})		c_m (nm^{-3})		*K*	
	L_o	L_d	L_o	L_d	L_o	L_d
O_2	0.0191 (±0.0033)	0.0096 (±0.0005)	0.0669 (±0.0008)	0.0624 (±0.0001)	3.49 (±0.72)	6.52 (±0.39)
water	30.6 (±0.0056)	28.0 (±0.0020)	6.071 (±0.006)	7.416 (±0.003)	0.1980 (0.0002)	0.2651 (0.0001)

Supplementary Figures

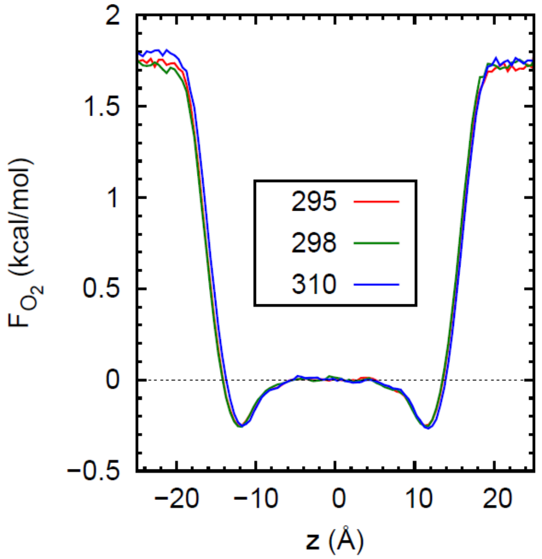

Supplementary Figure 1. Potentials of mean force for O_2 in a hexadecane slab (centered at z = 0) bounded by water slabs. PMF at 310 K from Ref. 4. All systems contained 10 O_2, 126 hexadecane and 2159 water molecules; those at 295 K and 298 K were simulated at constant temperature and surface area (NPAT)[11] using OpenMM[12]. Source data are provided as a Source Data file.

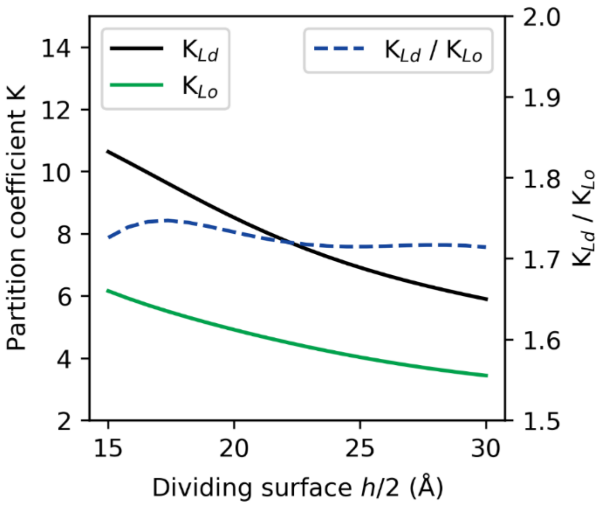

Supplementary Figure 2. Oxygen partition coefficients *K(h)* for the L_o and L_d phases (left axis) and their ratio (right axis, dashed line) as a function of membrane thickness *h*. Source data are provided as a Source Data file.

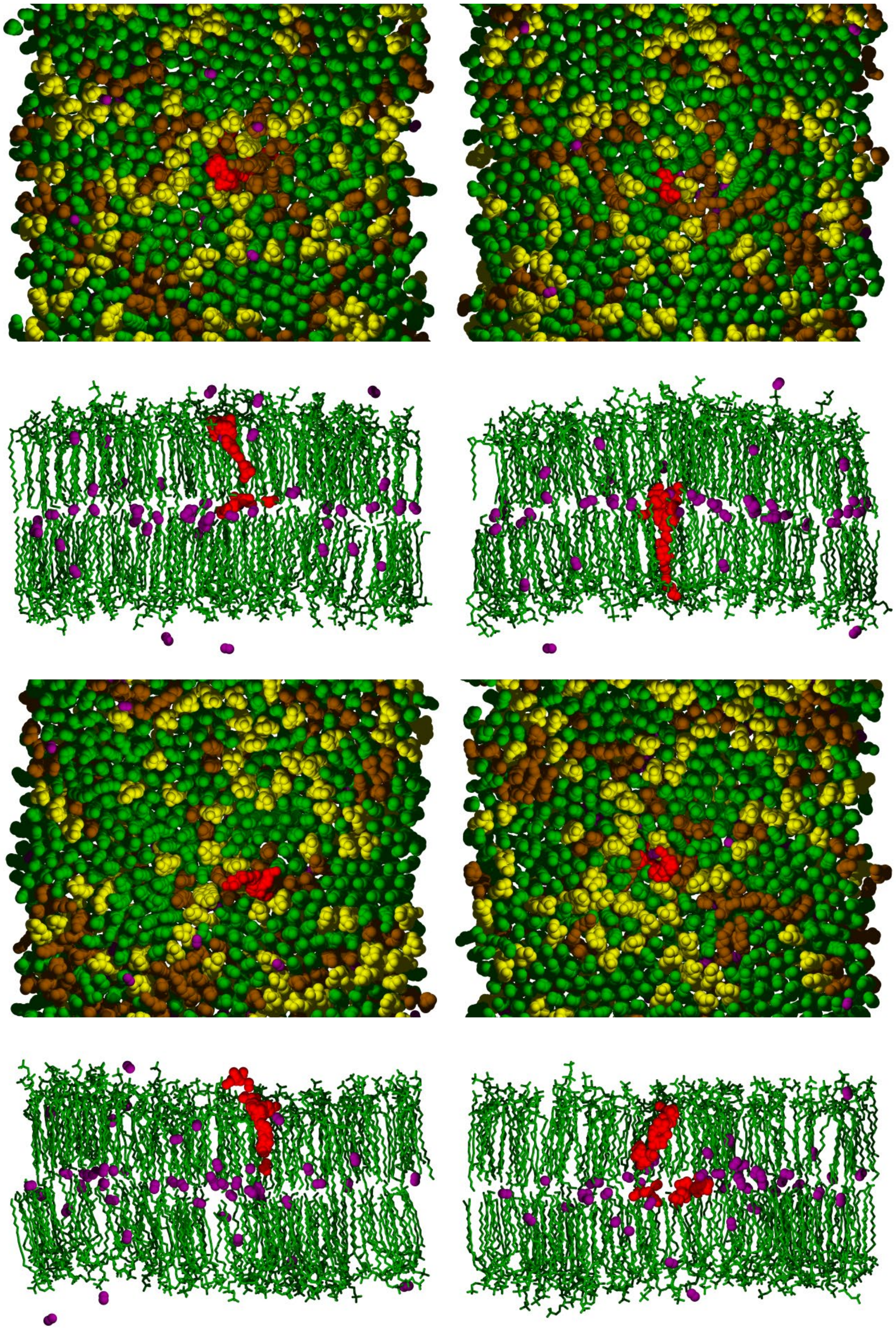

Supplementary Figure 3. Four additional simulation snapshots of a single oxygen (red) entering the liquid ordered phase spaced at 5 ps intervals over 500 ps. The bilayer is represented by the lipid configuration at the beginning of the time sequence, and waters and ions are removed in all panels. Top panel: a top-down view with headgroups removed to highlight the chain packing (DPPC green, DOPC brown, and cholesterol yellow). Bottom panel: side-view with DOPC and cholesterol removed, and with other oxygen molecules (purple); atoms in only half of the box are included for clarity.

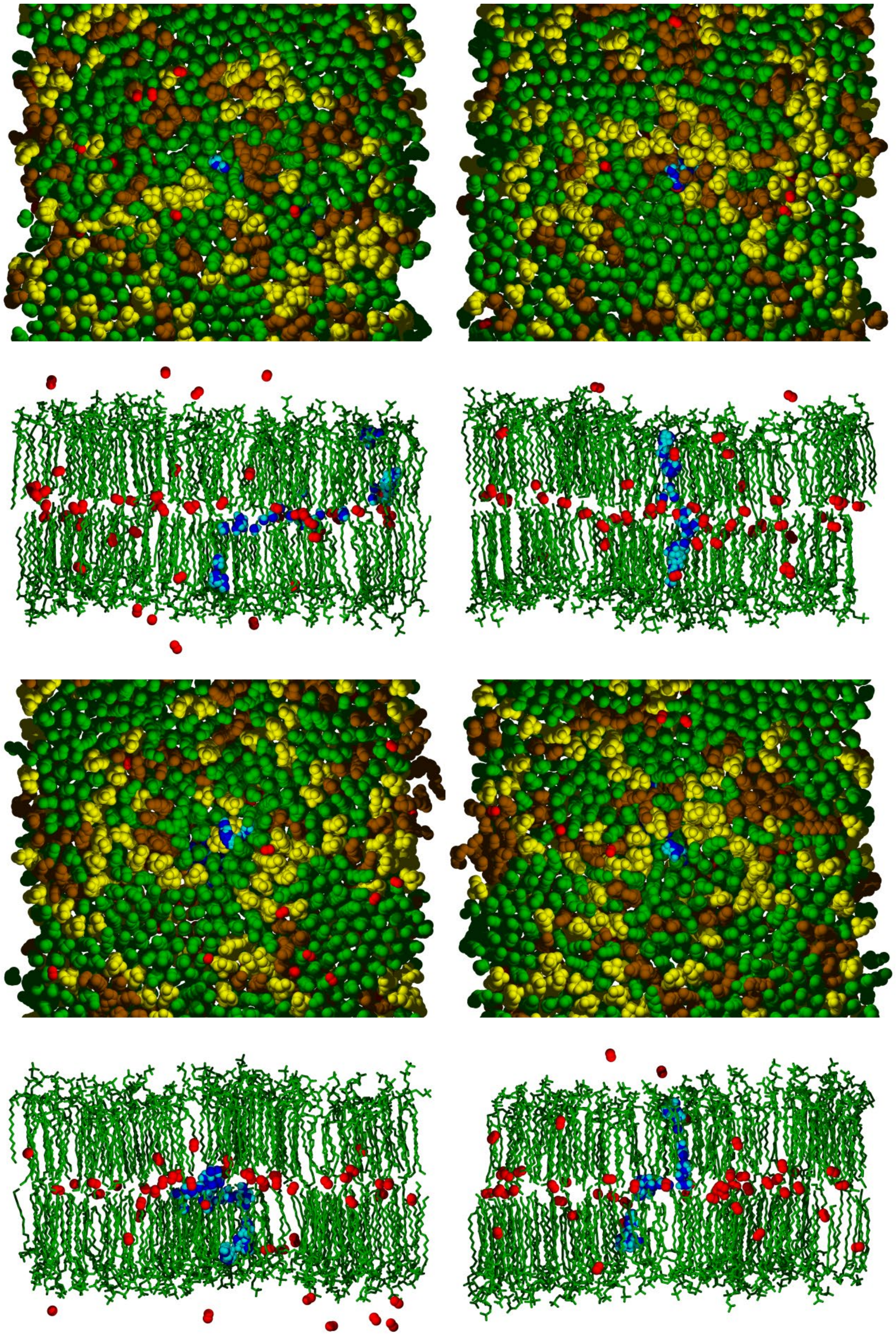

Supplementary Figure 4. Four simulation snapshots of a single water (blue oxygen, cyan hydrogen) entering the liquid ordered phase spaced at 5 ps intervals over 500 ps. The bilayer is represented by the lipid configuration at the beginning of the time sequence, and waters and ions are removed in all panels. Top panel: a top-down view with headgroups removed to highlight the chain packing (DPPC green, DOPC brown, and cholesterol yellow). Bottom panel: side-view with DOPC and cholesterol removed, and oxygen molecules in red; atoms in only half of the box are included for clarity.

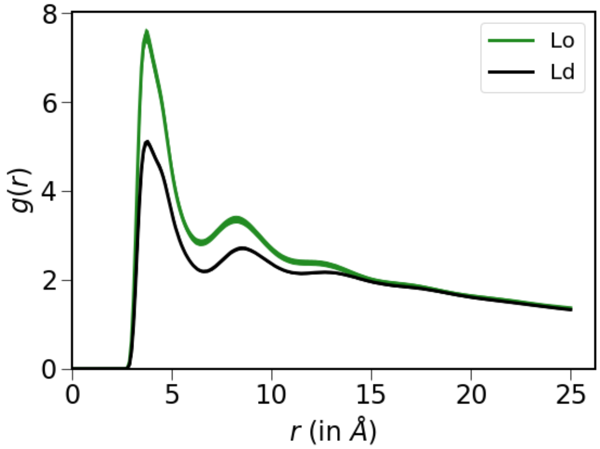

Supplementary Figure 5. Radial distribution functions *g(r)* between the methyl carbon of the *sn*2 chain of DPPC and oxygen atoms in the L_o and L_d phases. The thickness of the line is twice the standard error over 4 replicates (±2 ste). Source data are provided as a Source Data file.

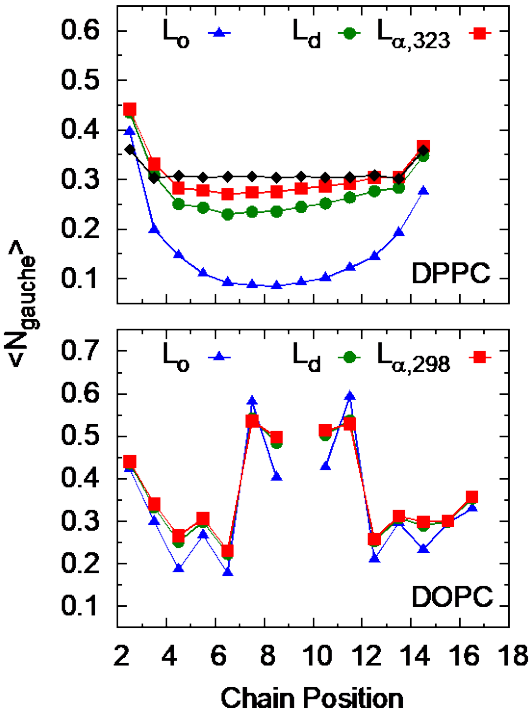

Supplementary Figure 6. Average number of gauche dihedral angles per dihedral angle for acyl chains of DPPC (top) and DOPC (bottom) for the L_o and L_d phases (at 298 K), and reference homogeneous fluid phase bilayers (DPPC at 323 K and DOPC at 298 K), and for hexadecane at 298 (top, black diamonds). Dihedral angles are labeled by the average position of the central two carbons (e.g., C1-C2-C3-C4 is 2.5). Results for DPPC in the L_o and L_d phases are not directly comparable to the L_α phase because of the temperature difference. Source data are provided as a Source Data file.

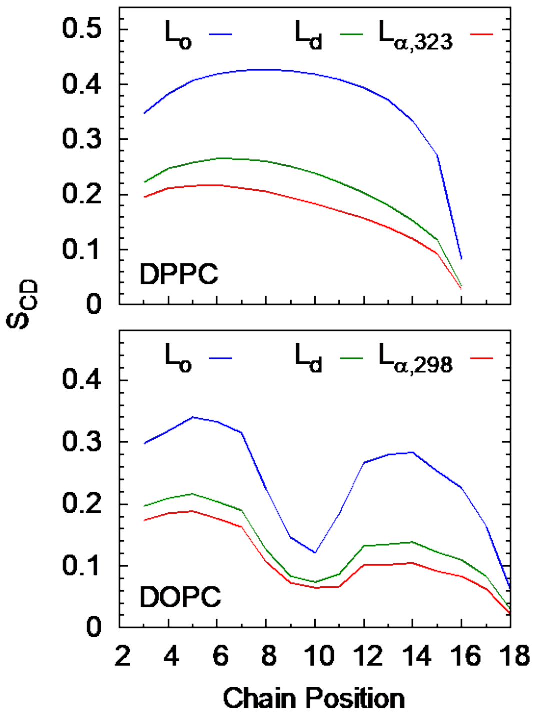

Supplementary Figure 7. Deuterium order parameters (S_{CD}) as for Supplementary Figure 6. Chain position refers to carbon number. Source data are provided as a Source Data file.

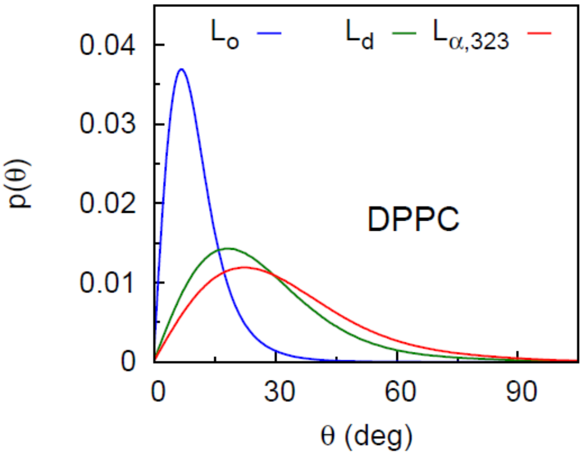

Supplementary Figure 8. Distribution of angles for DPPC chain vectors (calculated between carbons 4 and 14) with respect to the bilayer normal of the L_o and L_d phases (at 298 K), and reference homogeneous fluid phase bilayer $L_{\alpha,323}$ (DPPC at 323 K). Source data are provided as a Source Data file.

Supplementary References

1 Ju, L. K. & Ho, C. S. Oxygen Diffusion Coefficient and Solubility in n-Hexadecane *Biotechnol. Bioeng.* **34**, 1221-1224 (1989).

2 Moller, M. N. *et al.* Membrane "lens" effect: Focusing the formation of reactive nitrogen oxides from the (NO)-N-center dot/O-2 reaction. *Chem. Res. Toxicol.* **20**, 709-714 (2007).

3 Möller, M. N., Lancaster, J. R. & Denicola, A. in *Curr. Top. Membr.* Vol. 61 (ed Sadis Matalon) 23-42 (Academic Press, 2008).

4 Ghysels, A., Venable, R. M., Pastor, R. W. & Hummer, G. Position-Dependent Diffusion Tensors in Anisotropic Media from Simulation: Oxygen Transport in and through Membranes. *J. Chem. Theory Comput.* **13**, 2962-2976 (2017).

5 Altenbach, C., Greenhalgh, D. A., Khorana, H. G. & Hubbell, W. L. A Collision Gradient-Method to Determine the Immersion Depth of Nitrozides in Lipid Bilayers - Application to Spin-Labelled Mutants of Bacteriorhodopsin. *Proc. Natl. Acad. Sci. U. S. A.* **91**, 1667-1671 (1994).

6 Oh, K. J., Altenebach, C., Collier, R. J. & Hubbell, W. L. in *Bacterial Toxins: Methods and Protocols* Vol. 145 (ed O. Holst) 147-168 (Humana Press Inc., 2000).

7 Sodt, A. J., Sandar, M. L., Gawrisch, K., Pastor, R. W. & Lyman, E. The Molecular Structure of the Liquid-Ordered Phase of Lipid Bilayers. *J. Am. Chem. Soc.* **136**, 725-732 (2014).

8 Sodt, A. J., Pastor, R. W. & Lyman, E. Hexagonal Substructure and Hydrogen Bonding in Liquid-Ordered Phases Containing Palmitoyl Sphingomyelin. *Biophys. J.* **109**, 948-955 (2015).

9 De Vos, A. *et al.* Membrane permeability: characteristic times and lengths for oxygen, and a simulation-based test of the inhomogeneous solubility-diffusion model. *J. Chem. Theory Comput.* **14**, 3811-3824 (2018).

10 Venable, R., Krämer, A. & Pastor, R. W. Molecular Dynamics Simulations of Membrane Permeability. *Chem. Rev.* **119**, 5954-5997 (2019).

11 Zhang, Y. H., Feller, S. E., Brooks, B. R. & Pastor, R. W. Computer-Simulation of Liquid/Liquid Interfaces. I. Theory and Application to Octane/Water. *J. Chem. Phys.* **103**, 10252-10266 (1995).

12 Eastman, P. *et al.* OpenMM 7: Rapid development of high performance algorithms for molecular dynamics. *PLoS Comput. Biol.* **13,** doi:e100565910.1371/journal.pcbi.1005659 (2017).
